# Identifying functional groups among the diverse, recombining antigenic *var* genes of the malaria parasite *Plasmodium falciparum* from a local community in Ghana

**DOI:** 10.1371/journal.pcbi.1006174

**Published:** 2018-06-13

**Authors:** Mary M. Rorick, Edward B. Baskerville, Thomas S. Rask, Karen P. Day, Mercedes Pascual

**Affiliations:** 1 Department of Ecology and Evolution, University of Chicago, Chicago, IL, United States of America; 2 Department of Biology, University of Utah, Salt Lake City, UT, United States of America; 3 School of Biosciences, Bio21 Institute, The University of Melbourne, Melbourne, AU; 4 Department of Microbiology, New York University, New York, NY, United States of America; 5 The Santa Fe Institute, Santa Fe, NM, United States of America; ETH Zurich, SWITZERLAND

## Abstract

A challenge in studying diverse multi-copy gene families is deciphering distinct functional types within immense sequence variation. Functional changes can in some cases be tracked through the evolutionary history of a gene family; however phylogenetic approaches are not possible in cases where gene families diversify primarily by recombination. We take a network theoretical approach to functionally classify the highly recombining *var* antigenic gene family of the malaria parasite *Plasmodium falciparum*. We sample *var* DBLα sequence types from a local population in Ghana, and classify 9,276 of these variants into just 48 functional types. Our approach is to first decompose each sequence type into its constituent, recombining parts; we then use a stochastic block model to identify functional groups among the parts; finally, we classify the sequence types based on which functional groups they contain. This method for functional classification does not rely on an inferred phylogenetic history, nor does it rely on inferring function based on conserved sequence features. Instead, it infers functional similarity among recombining parts based on the sharing of similar co-occurrence interactions with other parts. This method can therefore group sequences that have undetectable sequence homology or even distinct origination. Describing these 48 *var* functional types allows us to simplify the antigenic diversity within our dataset by over two orders of magnitude. We consider how the *var* functional types are distributed in isolates, and find a nonrandom pattern reflecting that common *var* functional types are non-randomly distinct from one another in terms of their functional composition. The coarse-graining of *var* gene diversity into biologically meaningful functional groups has important implications for understanding the disease ecology and evolution of this system, as well as for designing effective epidemiological monitoring and intervention.

## Author summary

Many disease-causing microbes vary their surface proteins to escape their host’s adaptive immune response—a phenomenon called antigenic variation. Some such systems rely on large, hyper-variable multi-copy gene families that encode functionally diverse protein variants. Because these gene families typically diversify by recombination, standard methods for protein functional classification—which are based on the assumption of a simple, tree-like evolutionary history—are not possible. We take a network-based approach to the functional classification of the *var* multi-copy gene family, which encodes the dominant natural antigen of the malaria parasite *Plasmodium falciparum*. We do not assume a particular diversification process, or even that functional similarity correlates with sequence similarity. Our dataset comprises thousands of unique *var* types that we sampled from a population in Ghana, and we map this diversity to just 48 functional types. We find that coarse-graining *var* diversity into functional types reveals new patterns in the structure of the diversity, and these insights could help us better understand the mechanisms shaping the diversity of these genes. Furthermore, functional insight into *var* diversity can inform monitoring and intervention, including the possibility of a *var*-based malaria vaccine effective at preventing disease.

## Introduction

To address the functional variation present within variable gene families, phylogenetic analyses can typically identify structural and/or functional divergence occurring on a simple, tree-like evolutionary history (e.g., [1, 2]). However, this approach is not possible for protein families that have diversified primarily by reticulate evolution (i.e., recombination). Inferring reticulate phylogenies (a.k.a. phylogenetic networks) is notoriously difficult, involving NP-hard problems [3–5]. In this paper we present a method for describing the functional diversity of an ultra-diverse recombining gene family. The method does not require an inferred phylogenetic history or even an alignment of the full length sequences—key advantages when these are not available. We use this method to identify distinct functional types within the multi-copy antigenic *var* genes of the malaria parasite *Plasmodium falciparum* (*P*. *falciparum*). There are approximately 60 *var* copies per parasite genome, located in multiple subtelomeric and centromeric locations within the genome [6]. They encode the parasite’s primary natural antigen, *Plasmodium falciparum* erythrocyte membrane protein 1 (*Pf*EMP1). This large, multi-domain protein is expressed at the surface of infected erythrocytes (IEs), where it binds host endothelial receptors within the microvasculature to prevent IE circulation to the spleen, where infected cells are mechanically cleared [7]. Sequestration of IEs within host tissues is essential to parasite survival and underlies the unique virulence of *P*. *falciparum* relative to other malaria parasites of humans. *Pf*EMP1 is highly visible to the immune system and an important antibody target [8]. As a consequence, the parasite has evolved a system of antigenic variation to shift expression among the *var* copies such that only one is active at a time [9–11]. Certain *var* types are associated with particular receptor binding preferences, sequestration patterns within host tissues, and/or disease symptoms (reviewed in [12]).

*Var* diversity is extensive both within individual genomes as well as between parasite genomes. Thousands of distinct *var* genes can typically be sampled from areas of high endemnicity, with the majority of *var* sequences being unique between parasite genomes [13, 14]. *Var* genetic sequences have exceptionally low sequence identity due to ancient sequence divergence and strong antigenic selection. Not only is there a great deal of diversity in domain composition among distinct *var* sequences, but even within a single domain type, amino acid sequence identity is low (< 75% even for the most conserved domain, DBLα). Therefore, *var* domains are for the most part unalignable at the sequence level.

Despite this vast sequence diversity, it nevertheless seems unlikely that each *var* sequence variant is *functionally* unique. Functional classification of *var* diversity is of clinical interest for the purpose of designing effective monitoring and intervention (e.g., for studying the strain structure of the parasite [15], or for the possibility of developing a *var*-based vaccine [16]). However, mapping the vast number of *var* sequence types to a smaller number of meaningful functional types has remained a major challenge in the field.

Previous methods have succeeded in dividing up *var* type diversity into three groups based on upstream promoter sequence (*ups*) and chromosomal location: groups A, B and C [12]. Other network-based classification systems directly draw on the mosaic structure of this gene family and group *var* types based on the sharing of short sequence motifs, meaning that functional groups are defined as clusters in a recombination network [17–19]. The *var* recombination network appears to tightly correlate with *ups* classification. The earliest methods based on recombination networks used conserved sequence features within a single 100–150 amino acid tag within the only consistent extracellular domain, DBLα [20]. Most *var* classification methods are still based on this tag region. When full-length *var* sequence is available, the presence of entire conserved domain cassettes within the larger architecture of the protein can be used for functional classification [19]. *Var* functional groups based on sequence features of the DBLα tag appear to be largely congruent with groups that are based on larger portions of the protein sequence and/or the *ups* region [21]. Some *var* groups are associated with cytoadhesion traits implicated in severe disease symptoms, and/or they exhibit preferential expression in patients with these symptoms [12, 22–27]. A consistent finding has been that group A *var* genes tend to be expressed in patients with severe malaria (e.g., [23]).

A major motivation within the field currently is to pursue links between *var* geneotype and disease phenotype, in the hope that interventions—in particular vaccination—may be able to specifically target severe-disease associated *Pf*EMP1 functions [16]. In light of this goal, one limitation that all current *var* classification schemes share is that they assume similar sequences should have similar adhesive properties. However, this may not always be the case—especially for highly diverse protein families with reticulate histories. Being “one of most diverse adhesion modules in nature” [12], and undergoing constant recombination among these diverse lineages [6, 28, 29], the *var* gene family likely benefits from exceptionally efficient exploration of sequence space. It also experiences strong selection to simultaneously bind host endothelial receptors and evade specific immunity [30]. In this context, similar adhesive properties may evolve through convergence, and therefore have distinct ancestry. Due to the many-to-one nature of the genotype to phenotype map, non-homologous and dissimilar sequences may frequently share the same molecular function, and as a result, cause the same disease symptoms.

There is consensus that *Pf*EMP1 is characterized by a micro-modular structure and function. While the above classification schemes seek to uncover this structure by considering sequence similarity, we seek to uncover this structure by considering *function* specifically. We accomplish this by first breaking down the sequence diversity into its constituent recombining parts, or homology blocks (HBs), as described previously by Rask et al. [19]. We then functionally annotate these parts—not based on sequence similarity, but rather, based on a network approach that groups sequence parts sharing similar co-occurrence interactions.

More specifically, to functionally annotate the recombining sequence parts we use a flexible community detection approach to search for the optimal arrangement into groups. Groups are defined as elements that share similar interactions with other elements. Sharing similar interactions implies similar function within the larger network. We take a Bayesian approach and create a continuous analogue of a stochastic block model that we previously applied to identify trophic levels within food-web networks [31]. Here, we apply this method to the co-occurrence network of homology blocks sampled from approximately 10,000 unique *var* types within a highly endemic population in Ghana. We reason that homology blocks that have similar co-occurrence patterns with other groups of homology blocks can replace each other through recombination, and based on this we conclude that they likely share similar molecular function, and thus we define these groups as homology block functional groups—completely irrespective of whether there is shared ancestry or sequence similarity within groups. We use the functional annotation of homology blocks to redefine our ~10,000 *var* sequence types based on the homology block functional groups they contain. This allows us to substantially reduce the antigenic complexity of our dataset and gain new insights into the structure of *var* diversity in a highly endemic population.

Whether the immense sequence variation of the *var* genes map to a smaller amount of functional variation remains an open question with significant epidemiological implications [6]. In particular, *var* functional variation appears to play a role in determining disease severity (e.g., [23]), so even a coarse genotype-phenotype map of *var* gene diversity could greatly enhance our ability to combat *P*. *falciparum* through strain-specific vaccination or other targeted interventions [32, 33]. A simplified and functional understanding of *var* antigenic diversity could also allow for new, meaningful connections between *var* empirical data and several bodies of theoretical literature (e.g., [15]), which could in turn elucidate the dynamics shaping this lethal parasite’s epidemiology and evolution.

## Results

### Sampling *var* DBLα sequence tag diversity and identifying homology blocks

We collected a mean of 89.4 *var* DBLα sequence tags from each of the 209 isolates (standard deviation of 63.3, range from 5 to 375). As far as we know this represents the deepest *var* sampling to date. Among all 209 isolates we collected a total of 18,694 *var* sequence tags, which correspond to 11,385 distinct *var* sequence types after clustering into distinct *var* sequence types. The clustering is carried out to remove PCR-generated variation, but inevitably clusters some natural variation as well. For the majority of the isolates we also sampled the microsatellite allelic diversity at twelve loci. While these methods and results are primarily described in [34], here we used the microsatellite data to define single infection isolates as those with at most a single microsatellite allele at each of the twelve loci. There were 29 single infections by this criterion.

**Fig 1:**
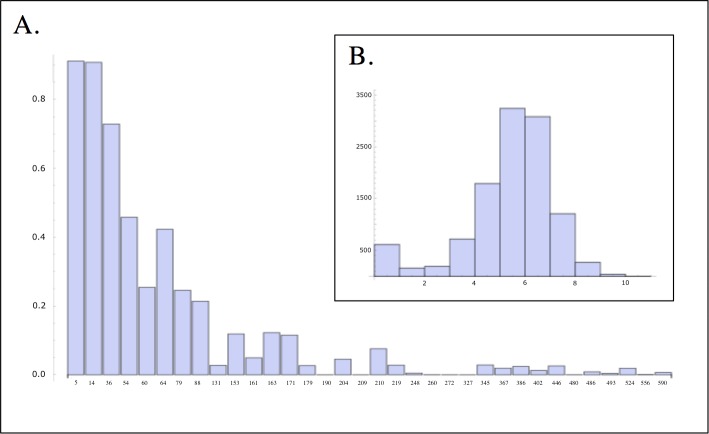
Among the 11,385 DBLα sequence tags, which correspond to unique *var* types: **(A)** The distribution of total HB counts per sequence tag. **(B)** The frequency of each HB in the entire dataset.

We translated all the sequences corresponding to a given *var* type, and performed a search for the homology blocks within each of the sequences [19]. We found 0–10 homology blocks per sequence (mean 4.9) (Fig 1). Taking a single sequence in the database as a representative for each *var* type, and excluding sites before the first homology block or after the last homology block, we found that an average of 89.3% of the sequence tag is covered by homology blocks. For each *var* type, we then took the mean presence/absence state for all homology blocks and for all sequences that map to that type. We then reduced the dataset to 9,588 sequence types through additional cleaning procedures based on the amino acid sequence and homology block match, to remove low quality sequences reflecting pseudogenes or regions outside of DBLα. Within this dataset there were 28 distinct homology blocks with a sufficient number of matches to be considered high quality (several additional homology blocks occurred only a few times, and we did not consider these further). HB 590, which occurred 51 times, was the rarest homology block that we considered. There were still some sequences with zero homology block matches in our dataset, and removing these reduced the dataset to 9,276 *var* types. Unless otherwise noted, this was the dataset we used for the following analyses.

### Homology block co-occurrences as a network

**Fig 2:**
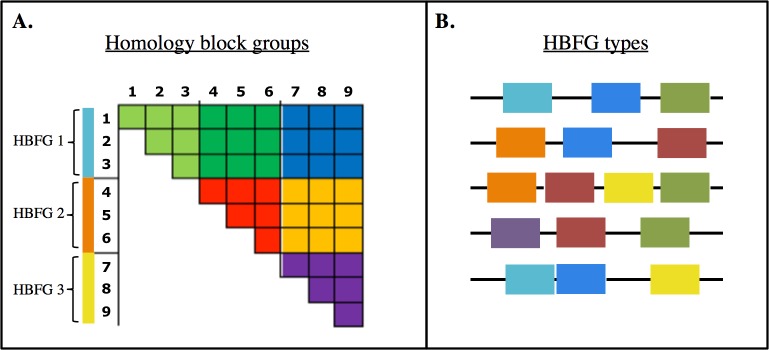
An approach to identifying functional groups within the multi-copy *var* genes. **(A)** Graphical representation of a stochastic block model for the normalized co-occurrence matrix **Z**, for nine homology blocks divided into three functional groups (HB functional groups 1–3). The blocks within the matrix of a single color represent the *z*-scores between pairs of HB functional groups, which are assumed to be drawn from the same normal distribution. **(B)** Homology blocks recombine to form *var* gene sequences, so after homology blocks are classified into functional groups (7 shown here, represented by distinct colors), *var* functional types can be defined as unique combinations of homology block functional groups (HBFG types). Viewing *var* sequences as HBFG types reduces diversity by over two orders of magnitude.

The method we use is based on an interaction matrix, **Z**, where entries (or *z*-scores) are deviations from a null model based simply on the frequency of the elements in the dataset. Fig 2 shows a simple example with three groups, each with three elements, arranged by group membership along the axes of the matrix. We assume *z*-scores between pairs of groups are drawn from the same normal distribution (represented as blocks of the same color in Fig 2). This means that we search for groups such that *z*-scores between pairs of groups (i.e., within blocks) will be similar. A flexible community detection method defines groups *not* as modules with strong within-group interaction, but rather *as elements sharing similar interactions with other groups of elements*—meaning that blocks of interactions within **Z**, with high or low interaction values, can appear off the diagonal of the matrix as well as along the diagonal. Modules, in contrast, would appear only as high interaction values within blocks along the diagonal of **Z**.

**Fig 3:**
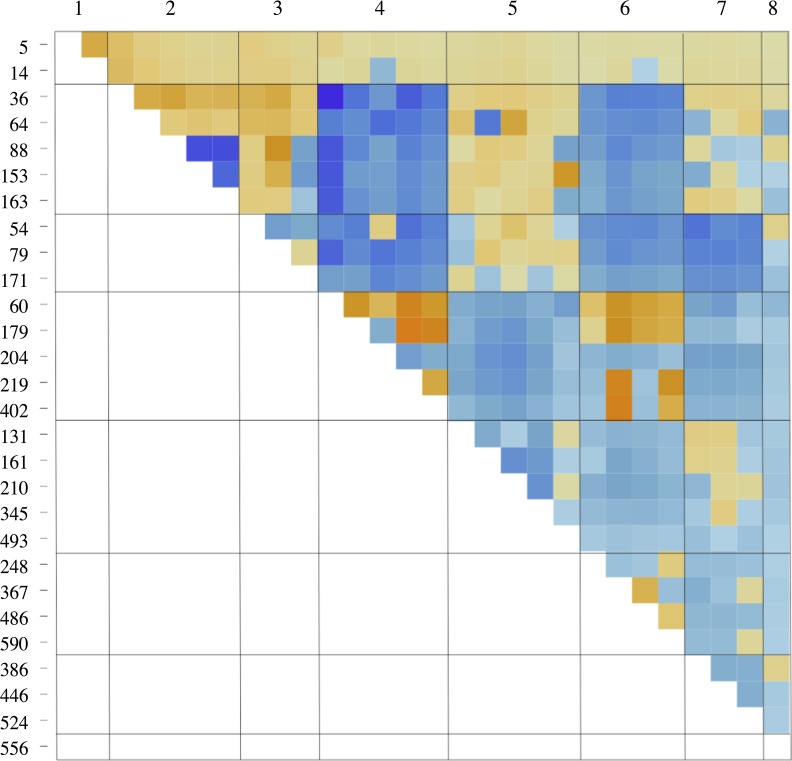
Best grouping of homology block linkage network according to Bayesian cross validation of the stochastic block model. Color represents the normalized co-occurrence score for each pair of homology blocks: colors toward blue represent more frequent co-occurrence than random; colors toward orange represent less frequent co-occurrence than random.

When considering our antigenic data as a network, the nodes are homology blocks and the edges/interactions are homology block co-occurrences within the larger protein sequence. When homology blocks have similar co-occurrences with other homology blocks, it means they are interchangeable with one another through recombination, which in turn implies that they have similar molecular function. We test for the existence of such functional groups of homology blocks by inferring the optimal community structure within our homology block co-occurrence network, and then testing whether the groups are functional-like as opposed to module-like. By searching for the optimal group arrangement based on a goodness of fit criterion we found that the 28 homology blocks are optimally arranged into 8 groups (Fig 3).

### Flexible community detection finds functional-like groups

Within the optimal group arrangement, group members share similar co-occurrence patterns with other groups, as evidenced by blocks of high (blue) and low (orange) interaction values in Fig 3. The fact that this best-fit arrangement into groups is not characterized by only high interaction values within blocks along the diagonal implies that the dominant community structure of this dataset is characterized by functional-like groups as opposed to module-like groups (Fig 3).

**Fig 4:**
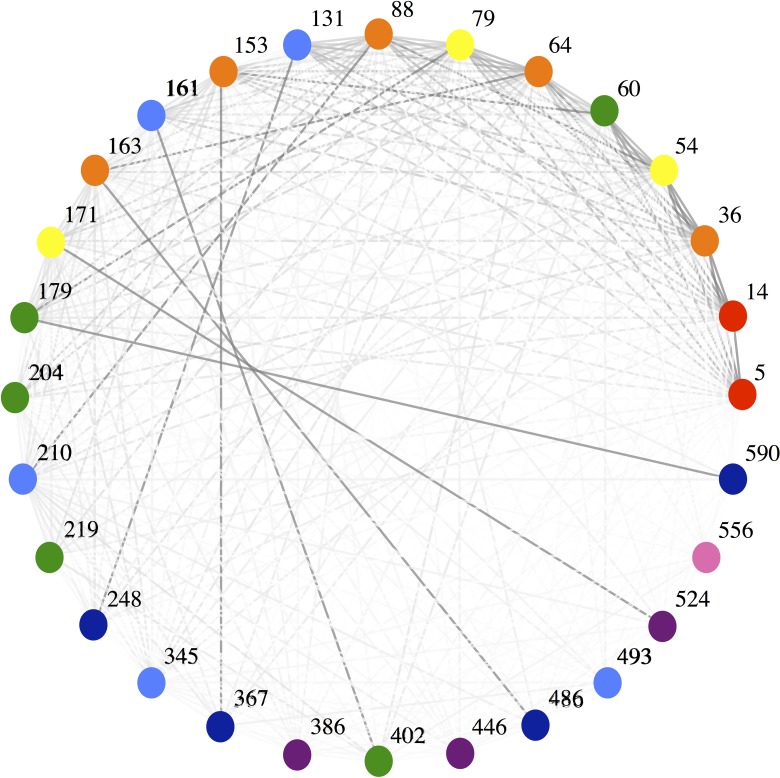
Network of the connectivity between the 28 HBs within the set of 9,588 *var* types, correcting for node degree. Colors of nodes show the eight distinct HB groups. Connectivity is defined as the number of connections between two HBs (co-occurrences) divided by the mean number of total connections for both HBs in the pair. Plotted with the Mathematica (v.10) “Graph” function using the circular embedding method.

The functional-like nature of the optimal group arrangement is also apparent in the interaction network of homology blocks, after correcting for node degree and coloring nodes by group membership (Fig 4). We can see that the connectivity is generally stronger between homology block groups rather than within groups. These results imply that our method has successfully found functional-like groups of homology blocks, and so we will henceforth refer to these eight groups as homology block *functional* groups, or equivalently, HB functional groups.

### Homology block overlap

Identifying homology block functional groups is not a trivial outcome of there being overlap among homology blocks within the *var* sequence tags (something that is allowed with our HB matching rules, and observed to some extent in our results). For one thing, the community detection method we use is not designed to group homology blocks based on high co-occurrence. Furthermore, as described above, we observe that homology blocks within groups have low co-occurrence relative to homology blocks between different groups (Figs 3 and 4). In fact, as discussed further below, we find that homology blocks within groups often have similar sequences and/or locations within the sequence tag—but this occurs *despite* them having relatively low co-occurrence within sequence tags. The pattern of low co-occurrence among homology blocks within groups is consistent with the idea that these groups describe sets of functionally redundant homology blocks.

### Validating functional groups with evidence of shared ancestry

Functional similarity can be a consequence of shared ancestry and conserved function. Therefore, even though we do not infer homology block functional groups based on sequence similarity or location similarity, we nevertheless expect it to be common for homology block sequences with similar function to have common ancestry, and thus, detectable similarity in sequence or location. We use this assumption to validate our functional groups—i.e., by looking for conservation of sequence identity and/or location that statistically exceeds the random expectation.

We examine the homology block sequences and their positions within the larger protein sequence tag to determine whether homology block functional group members show evidence of shared ancestry. We consider whether homology blocks within functional groups have similar sequences with respect to amino acid identity, and similar locations within the context of the larger protein, relative to the expectation for random homology block pairings within our sample.

For this analysis we only consider the six HB functional groups that are composed of more than two homology blocks (i.e., we exclude from the analysis HB functional groups 1 and 8). Group 1 is composed of only HB 5 and HB 14. Because both of these homology blocks are nearly always present in DBLα, they share the same pattern of co-occurrence with all other homology blocks, and so this is likely why these two homology blocks are grouped together. Group 8 only contains HB 556, so comparisons cannot be made between homology blocks within this functional group.

**Fig 5:**
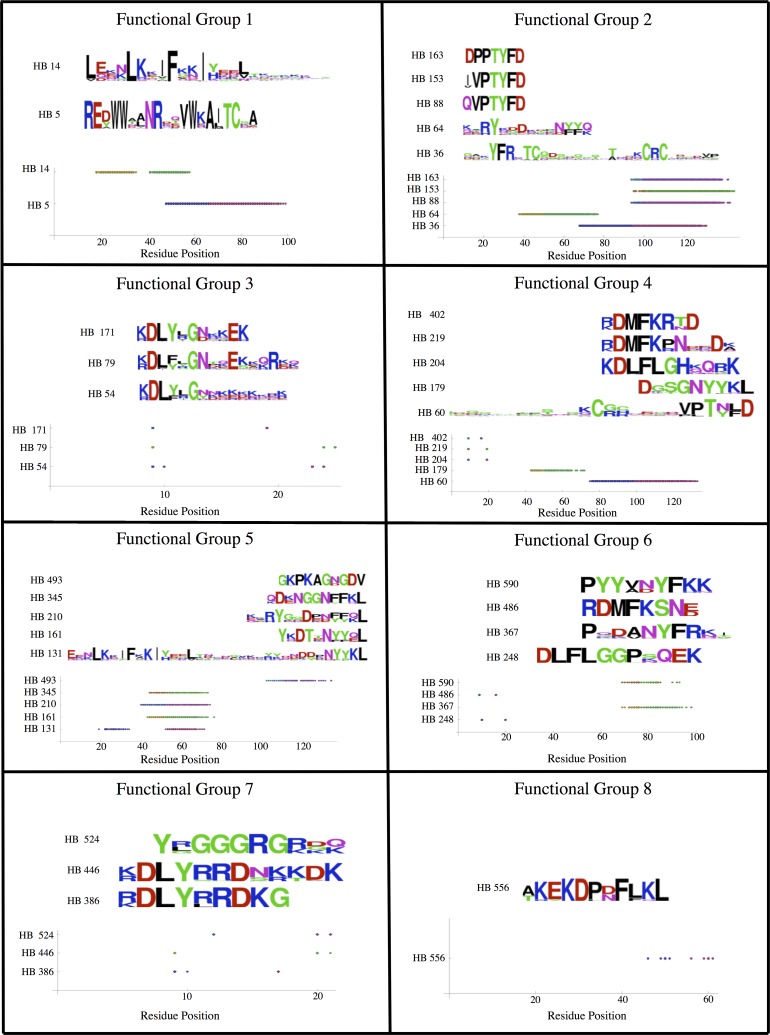
The sequences and locations of the HBs for each of the HB functional groups. The sequences are depicted with Logos in the top portion of each panel. The location(s) of the HBs within each of the sequence tags are depicted in the lower portion of each panel, with first and last positions in distinct colors.

We find more sequence similarity among homology blocks within functional groups as compared to between functional groups (Fig 5). Statistically, homology block sequences within HB functional groups are significantly more similar to one another than the random expectation (p = 0.0015–0.03, depending on the sequence similarity metric we use). We also analyzed the location of the homology blocks within the sequence tag to look for signs of conserved location within the functional groups. For all groups, members were primarily located in only one or two regions of the tag (Fig 5), and we find that the homology block’s location within the larger protein sequence tag is conserved within functional groups beyond the random expectation (p = 0.013).

### Classification based on homology or recombination

The *var* functional groups we infer are not the same as *var* groups that would be inferred based on sequence similarity (homology). Although we observe significant sequence and location similarity within the homology block functional groups, there is not consistent conservation of either homology block sequence or homology block location within HB functional groups. Many homology block sequences within HB functional groups do not share any apparent sequence or location similarity (Fig 5). The homology block functional groups described here are very different from what would have been created had we merely aggregated homology blocks based on the similarity of their underlying HMMs, or based on the similarity of the sequences and/or sequence locations to which they map.

**Fig 6:**
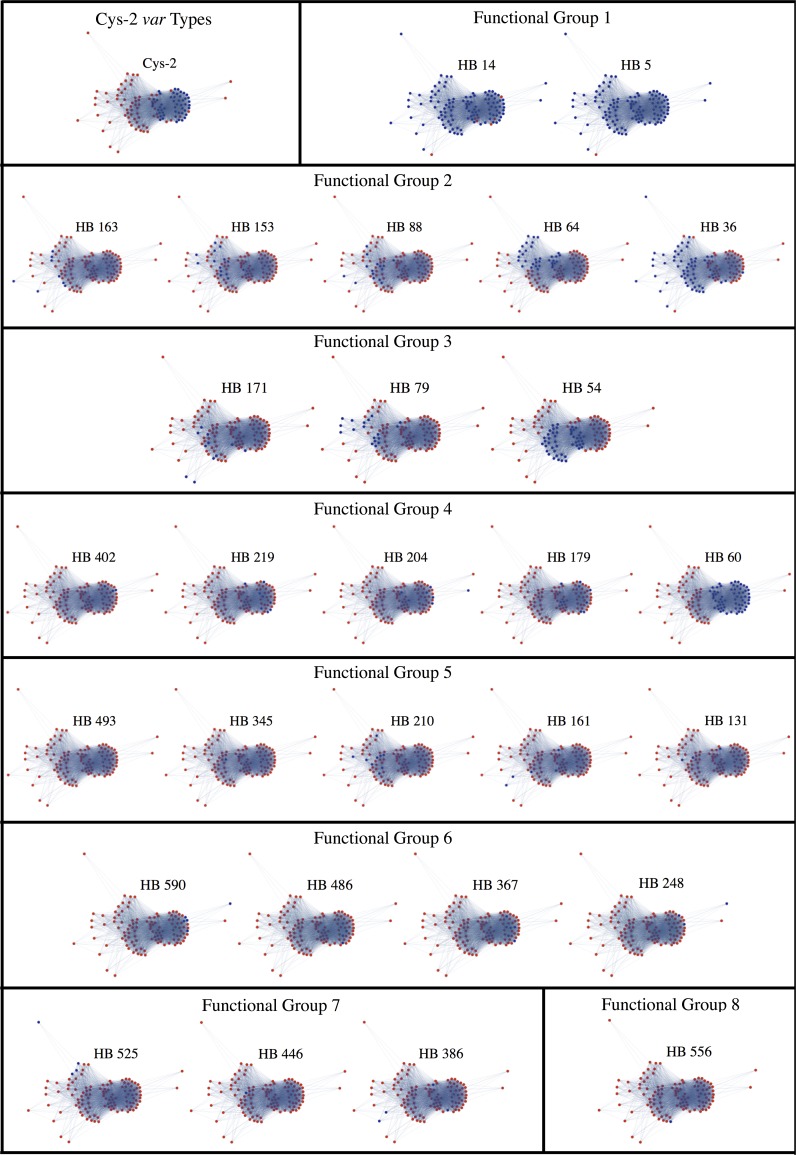
The relationship between the *var* homology block recombination network, cys-2 *var* genes, and the *var* genes containing each homology block functional group. All panels show the recombination network, as defined in Materials and methods. Panel titles indicate which nodes are shown in blue. Remaining nodes are shown in red.

The *var* functional groups we infer are also not equivalent to *var* recombination groups. The homology block functional groups do not appear to correspond strongly to the modular structure of the *var* type recombination network. Homology block functional groups only cluster within the *var* recombination network insofar as the homology blocks within a given functional group are generally either associated with cys-2 *var* types or non-cys-2 *var* types (and the distinction between these two *var* types is reflected strongly in the recombination network) (Fig 6). Beyond this, however, there appears to be no clustering of homology block functional groups within the recombination network of *var* types (Fig 6). This finding implies that these groups cannot be alternatively inferred by simply considering the modularity of the *var* type recombination network and the homology blocks within each *var* type. Rather, the inference of these groups seems to require identifying groups of homology blocks that share similar co-occurrence interactions with other homology blocks.

### Coarse-graining antigenic diversity

We use the functional annotation of homology blocks to simplify our antigenic dataset into a smaller number of biologically meaningful types. We find that there are only 882 unique combinations of the 28 homology blocks among the 9,276 *var* types within our dataset. Furthermore, when we map the 28 homology blocks to the eight homology block functional groups, we can simplify the picture further. If any member of a given homology block functional group is present in a sequence, then that group is considered present. Using these presence/absence states we can then classify *var* sequences as unique combinations of HB functional groups, which we call homology block functional group types (HBFG types). Within our entire dataset of 9,276 *var* sequence types, we find that there are only 48 HBFG types, or in other words, 48 *var functional* types. The coarse-graining of *var* diversity into only 48 functional types allows for isolates and antigenic types to be more comparable in number. This simplification can in turn be useful for analysis, visualization and comparisons between field-collected data and theory.

### Functional distribution of antigenic types in isolates

**Fig 7:**
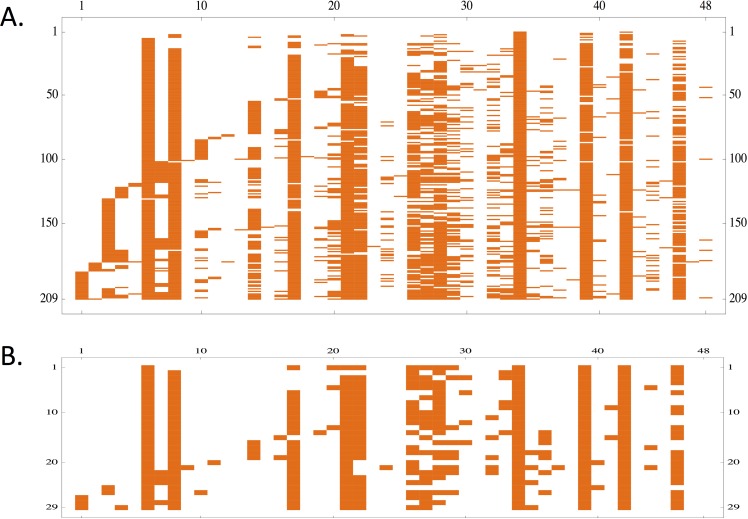
**(A)** The 48 distinct HBFG types are ordered along the horizontal axis by similarity, based on their HB presence/absence profile. The 209 isolates are ordered along the vertical axis by similarity, based on their HBFG type presence/absence profile. The elements on both axes are sorted by the Mathematica function “Sort”, which places elements in canonical order. **(B)** The same as above, but for the 29 single infection isolates only.

With this new view of *var* diversity, we look for insights into how *var* functional diversity is distributed within parasite genomes and populations. At the resolution of HBFG types, *var* diversity no longer appears as a chaotic cloud, with most variation being incomparable between different isolates. Fig 7 shows how the 48 HBFG types occur in the 209 isolates and in the 29 single infection isolates. One clear pattern that emerges is the presence of regularly spaced stripes along the axis of HBFG type similarity. This pattern reflects that common HBFG types are non-randomly distinct from one another.

A possible explanation for this is that *Pf*EMP1 proteins with similar HB profiles may be functionally redundant, and therefore may not be useful within the same parasite genome or within-host environment. Furthermore, antigenic sequences comprising similar functional parts may be antigenically similar as well. Thus, common HBFG types may select against intermediates with redundant function because they compete antigenically via the host immune response.

The pattern in Fig 7 is a form of nonrandom structure in the functional composition of *var* types within this population. This form of nonrandom structure may be related to other forms of nonrandom structure within the distribution of *var* types in this population, which we have reported previously [35]. Larger field-sampled datasets of *var* diversity collected in subsequent studies of this population may in the future be able to address the possible connection between these different forms of non-random structure within the *var* antigenic types of this population.

## Discussion

From a local population in Ghana we sampled the highly diverse antigenic *var* gene sequences of the malaria parasite *P*. *falciparum*. We decomposed this diversity into its recombining parts, and identified 28 homology blocks that recombined to form the observed sequence variants. We then asked whether some of these homology blocks are functionally redundant. Functional groups comprise elements that share similar interactions with other groups. Unlike modules, functional groups have more interactions between them than within them. Members of a given functional group can be considered functionally redundant. While modules of *var* sequences may be the result of overlapping HBs, or physical linkage among some *var* types, or other features of the *var* recombination hierarchy, functional groups of *var* sequences offer insights into the functional differences among *var* types. We find that the 28 homology blocks can be broken down into just eight functional groups.

We observed limited, yet statistically significant signals of shared ancestry within homology block functional groups (i.e., conservation). This finding proves that the method we use—which does not define functional groups based on homology—identifies biologically meaningful functional groups. However, not all sequences within a functional group have clear sequence similarity, or map to the same region of the tag. We would not have identified these functional groups using only signals of conservation. Our method can be used for the inference of common function even when sequence similarity does not exist, either due to divergence or distinct origination (i.e., convergent evolution)—phenomena that are especially likely in the *var* genes, as they are characterized by architectural flexibility and extreme recombination rates.

While homology blocks within functional groups exhibit a significant degree of conservation of location within the larger sequence tag, there is nevertheless considerable variation in location within most HB functional groups. This variation suggests a high degree of functional modularity since a homology block sequence from a given homology block functional group can apparently perform its molecular function similarly in diverse protein structural contexts. Functional modularity has been demonstrated for *var* proteins at larger structural scales, such as protein domain function and organization, so it is interesting that we see evidence for this also at the very fine spatial scale of the HB.

Our functional categorization of *var* sequence diversity allows us to describe a set of antigenic types that is more than two orders of magnitude less diverse than the original antigenic dataset. In a dataset of 18,694 sequences and 9,276 distinct sequence types, there are only 882 distinct combinations of HBs and only 48 distinct combinations of HB functional groups. We find that this simplification provides new insights into the functional diversity of *P*. *falciparum var* genes. Specifically, we observe a pattern that indicates that the most common HBFG types (*var* functional types) are non-randomly distinct from one another with respect to their HB functional group composition.

Our functional description of *var* diversity also serves to increase levels of *var* repertoire overlap from close to zero to more measurable levels. Distinct isolates become more comparable when there is significant overlap among their antigenic repertoires, and the distribution of overlap indices within the population as a whole becomes more interesting. The overlap that exists among distinct antigenic types circulating in a local population is a critical feature for studying certain epidemiological and ecological dynamics [35]. HBFG types may therefore be useful for future studies of these dynamics.

Simplifying antigenic diversity into distinct functional groups provides insight into the actual number of functionally distinct antigenic units circulating in a local population. It is the antigenic *functional* diversity, as opposed to the antigenic sequence diversity, that is expected to shape this parasite’s epidemiology and evolution. A meaningful quantification of *var* functional diversity could facilitate theoretical progress in the field. A long-term goal should be to understand this functional diversity, how it interacts with the host immune system, and how it evolves.

Parsing the immense natural variation of *var* sequences into meaningful functional categories could have major implications for monitoring, control and treatment of malaria. Some *var* sequences have been linked to severe disease, while others appear benign. For example, the evolution and transmission of virulent antigenic functional groups could be specifically monitored—saving valuable public health resources. Similarly, identifying a limited set of disease-causing antigenic functional groups would greatly advance the possibility of someday developing a multivalent vaccine with high efficacy at preventing disease.

The approach we take here for the functional annotation of *var* antigenic sequences may in the future be useful for the functional annotation of other ultra-diverse gene families beyond the *var*. Other protein families mediate interactions between multiple species, are under strong diversifying selection, and encode diversity within individual genomes via multi-copy gene families. Moreover, it is normal for multi-copy gene families to diversify by extensive and frequent non-allelic recombination. Examples include the vertebrate major histocompatibility complex (MHC) genes and the genes encoding the variant surface glycoproteins (VSGs) of the African sleeping sickness parasite, *Trypanosoma*. Our approach might also be useful for decoding the functional diversity of ancient gene families that are so highly diversified at the sequence level that meaningful sequence alignment becomes difficult (consider for example the ribosomal protein L36 for which only a single amino acid residue is conserved in indisputable homologs [36]). In sum, our approach may be useful for the functional understanding of any set of sequences with a complex evolutionary history because these methods do not rely on the assumption of a simple, tree-like bifurcating process dominating.

## Materials and methods

### Ethics statement

As this study involved human subjects, IRB approvals were obtained prior to data collection and analysis at the authors’ institutions. IRB approval numbers for this study are as follows (NYU and Michigan now closed due to transfer): NYU: S12-02449; UniMelb: HREC 1441986; Michigan: HUM 00078673; Chicago: IRB14-1495; Navrongo: NHRC IRB-131; Noguchi: CPN 089/11-12. Methods were compliant with ethical practice standards, including that informed consent was obtained from human subjects prior to their involvement.

### Study site and genetic sampling

Our dataset is a sample of *Plasmodium falciparum var* antigenic sequences collected at the end of the dry season in Bongo District (BD), Ghana. Details on the study design, study population and data collection procedures have been described previously [34]. Sampling was carried out across two broad catchment areas—Vea/Gowrie and Soe. Only *P*. *falciparum* positive samples identified by microscopy were used for molecular analysis. *Var* DBLα tags were sequenced for 209 *P*. *falciparum* positive samples. Twelve microsatellite loci were also sequenced for the majority of these isolates, as described in detail in in [37]. The multiplicity of infection (MOI), which is the number of parasite genomes per sample, was estimated as the maximum observed number of microsatellite alleles per locus. Single infection isolates were thus defined as those with at most one microsatellite allele at every microsatellite locus.

It is not yet technically possible to include *var* genetic diversity in studies of genome-wide variation in *P*. *falciparum* [38–41]. Studies of *var* diversity in the field still rely on sequencing a molecular marker with degenerate primers: a 100–150 tag sequence within DBLα—the only domain found in nearly all *var* genes [20, 42–45]. We sequenced the entire length of the PCR amplicon without the need for assembly. We assigned DBLα sequences to *var* types in a manner consistent with the 96% nucleotide identity definition commonly used in the field [42]. Clustering at this threshold is conservative, in order to ensure that distinct *var* sequence types represent natural variation as opposed to sequencing errors (thus, some of the sequence variation within a *var* type represents natural variation, which is ignored in this analysis).

All analyses were run using Mathematica v8 scripts unless otherwise noted. We translated DNA sequences to amino acid sequences using the software program EMBOSS Transeq [46, 47]. We excluded from the analysis sequences that had an unexpected reading frame, apparent frame shift substitutions or stop codons.

### Homology block composition of sequence tags

We identified homology blocks within our dataset of *var* sequence tags using the VarDom webserver, with a gathering cut-off of 9.97 to define a match [19]. Homology blocks are defined by hidden Markov models (HMMs) [19]. As such, they have a flexible length, and each amino acid position along their length is flexible, with the chemical properties of amino acids being considered implicitly through the HMM transition probabilities. The extent to which the homology block sequence is conserved in both sequence composition and length is described by the HMM. These homology blocks are distinct from the DBL “blocks” of Bull et al. [17], which have a rigid sequence length of 4 amino acids, and a completely rigid sequence identity. The homology blocks of Rask et al. are also distinct in definition from the DBL “homology blocks” of Smith et al. [20]. Smith et al. describe DBLα as being decomposable into a set of semi-conserved regions alternating with hypervariable regions, each of which is termed a homology block. In practice there is minimal overlap between the homology blocks of Smith et al. and those of Rask et al.

### Community detection method

We use the homology block composition of *var* sequence types to build a network of homology block co-occurrence. We use a continuous analogue of a discrete-valued stochastic block-model to infer the optimal arrangement of homology blocks into groups, with groups being defined as having similar interactions (co-occurrences) with other groups of homology blocks. The algorithm searches for the optimal group arrangement such homology blocks within groups share similar interactions with other homology blocks. This is a very different criterion to maximizing within-group connectivity (i.e., to identify modules). We account for variation in node degree within our null model, so our community detection method will not cluster homology blocks just because they have similar frequency within the dataset.

We define a real-valued matrix **Z**, which measures the frequency of co-occurrence of each pair of homology blocks relative to a null distribution in which homology blocks co-occur randomly according to their observed individual frequencies. We assume that, for each pair of groups, the entries in the matrix **Z** are drawn from a common normal distribution. This model is a continuous analogue of discrete-valued stochastic block model that has been used previously to describe network data [31, 48, 49].

More specifically, each entry *z*_*ij*_ of the matrix **Z** is equal to (*n*_*ij*_−*m*_*ij*_)/*s*_*ij*_, where *n*_*ij*_ is the number of samples where homology blocks *i* and *j* co-occur; and *m*_*ij*_ and *s*_*ij*_ are the expectation and standard deviation of the number of samples where *i* and *j* would co-occur, assuming an independent Bernoulli model based with the observed individual frequencies. Specifically, *m*_*ij*_ = *Np*_*i*_*p*_*j*_, and *s*_*ij*_^2^ = *Np*_*i*_*p*_*j*_(1 –*p*_*i*_*p*_*j*_), where *N* is the total number of observations and *p*_*i*_ and *p*_*j*_ are the observed individual frequencies of *i* and *j*.

We use Bayesian leave-one-out cross validation to evaluate the goodness of fit (*GF*) of any particular arrangement into groups. For a particular unordered pair of groups *gh*, we measure the goodness of fit of the corresponding entries in the matrix as $l_{gh}=\sum_{ij}{\text{E}}_{\backslash ij}^{\text{post}}[\log p(z_{ij})]$: the sum of the posterior expectation of the log-probability of each entry *z*_*ij*_, leaving out that entry when computing the posterior distribution. This means we measure the goodness of fit of a pair of groups (a block within **Z**) as the sum of the posterior expectations for the log-probabilities of the *z*-scores within the block. It also means that we cross validate by estimating the parameters that govern a normal distribution from which a particular entry (*z*-score) is drawn while leaving out that entry. The mean μ and precision τ for each block *gh* are assigned a normal-gamma prior distribution, where τ is gamma-distributed with shape 0.5 and rate 0.5, and, conditional on τ, μ is normally distributed with mean 0 and precision 0.5τ. The prior distribution (for the parameters that govern the normal distribution) is assumed to be normal-gamma, for convenience, because this is conjugate to the likelihood (the normal distribution describing the conditional probability of our entries given a particular μ and τ). The fact that the prior is conjugate to the likelihood means we already know the analytical form of the posterior (it has the same analytical form as the prior, just with new parameters). This means, in order to estimate the leave-one-out posterior expectation of the log-probability for each entry in the matrix, we can sample the posterior for μ and τ directly. The total goodness of fit for a particular arrangement into groups is then just $GF=\sum_{gh}l_{gh}$, the sum of the goodness of fit measures for each pair of groups (i.e., blocks within **Z**).

For computational efficiency, we use greedy agglomerative clustering to find the best arrangement into groups. If the total number of groups is equal to *k*, and the total number of elements being grouped is equal to *N*, we start with each homology block assigned to its own group such that *k* = *N*. We calculate the *GF* for this arrangement *G*_*k*_. We then see which pairing of two groups gives us the best improvement in *GF*, and we make that new pairing, to produce a new arrangement *G*_*k* = *N*-1_. We then continue to combine groups until we no longer improve the fit, or for a complete hierarchical clustering, until all elements are within a single group and *k* = 1. The code that implements this procedure is provided in the Supporting information (S1 CodeAndData), and an outline of the algorithm follows below:

1. Assign each item *i* of *N* items to its own group, and name this assignment *G*_*N*_.
2. Calculate the goodness of fit *GF*(*G*_*N*_).
3. Repeat with *k* = *N– 1* to *k = 1*:
  - For each pair of groups *gh* in assignment *G*_k+1_:
    - Combine *g* and *h* into a single group to form assignment *G*_*k*_^*(g+h)*^
    - Calculate the goodness of fit *GF*(*G*_*k*_^*(g+h)*^)
  - Set *G*_*k*_ equal to the assignment *G*_*k*_^*(g+h)*^ with the best goodness of fit *GF*(*G*_*k*_^*(g+h)*^)
  - If *GF*(*G_k_*) < *GF*(*G_k+1_*), terminate.

Upon termination, the best grouping *G* is the one with the best goodness of fit *GF*(*G*). This clustering algorithm will not necessarily identify a global maximum in *GF*; at the expense of computational efficiency, a non-greedy search algorithm such as simulated annealing could be used instead.

### Assessing similarity between distinct homology blocks

In this study we test for sequence similarity among distinct homology blocks. We also test for similarity between homology blocks with respect to their location with the larger sequence tag. To distinguish the two, we use the term “sequence similarity” to mean considerations of similarity with respect to amino acid identity, and “location similarity” to refer to similarity with respect to homology block location within the larger sequence tag.

We assess homology block sequence and location similarity qualitatively by visualizing the homology blocks as logos [50] (Fig 5). To generate each logo we use the sequence variation within our dataset that matches a given homology block. We also statistically test whether sequence similarity within homology block functional groups is greater than expected at random, given the set of 28 homology blocks in our dataset. We compare the sequences of distinct homology blocks by comparing their consensus sequences within our dataset, which is defined as the most common amino acid state at each position along the homology block sequence, given the sequence variation in our dataset that corresponds to a given homology block. We use two different indices of similarity to compare pairs of consensus sequences: Needleman Wunsch (NW) similarity and Smith Waterman (SW) similarity. While it is unconventional to use metrics other than Hamming distance to compare genetic sequences, NW similarity and SW similarity are more appropriate and informative than Hamming distance for our purposes, because we need to be able to meaningfully compare sequences of different length, often without clear homology.

We assess location similarity between distinct homology blocks by considering the distance (in amino acid residue positions) between their consensus start positions. Homology block start positions are defined with respect to their start position within the DBLα tag. The DBLα tag, in turn, has a consistent start site within the larger DBLα domain because it is characterized by one of the only consistent and highly conserved amino acid motifs in the entire extracellular portion of *Pf*EMP1 [42]. We qualitatively assess similarity between homology blocks by plotting the start positions of a homology block in each of the sequences within our dataset (Fig 5). We also statistically test whether the distance between the start sites of pairs of homology blocks is on average smaller within functional groups than the expectation for random pairs of homology blocks.

We use a randomization procedure to statistically test whether there is significantly greater conservation within homology block functional groups than expected randomly given our complete set of 28 homology blocks. The random expectation is expressed as a null distribution generated by taking samples of pairs of homology blocks from all 378 possible pairwise comparisons, without replacement. Random samples are the same size as the observed sample of pairwise comparisons (N = 42). The observed average index (for sequence similarity or distance between start positions) is then simply compared to the distribution of averages from 100,000 random samples. This generates a one-tailed p-value for the observed, within-functional-group index.

### Mapping functional groups onto the *var* recombination network

To address whether homology block functional groups reflect *var* recombination groups, and in particular the cys-2 *var* gene distinction, we first created a recombination network of *var* genes (Fig 6). We accomplished this by representing the *var* DBLα sequence types as nodes, connected by edges representing historical recombination events. For this visual analysis we only consider the DBLα types that occur within the single infection dataset more than once—a very restrictive criterion that dramatically reduces the number of sequence types, and thus, the size of the resulting network. We connect nodes with an edge when the DBLα types share any homology block other than the three most pervasive ones (HB 5, HB 14 and HB 36) that are present in >50% of the sequences. After constructing this *var* recombination network, we map cys-2 *var* genes onto it. One of the most robust functional groups among *var* types that has been described previously is the cys-2 group, and these *var* types can be inferred directly from the DBLα tag region based on the number of cysteines within the tag (i.e., by the presence of exactly two cysteines within the DBLα sequence tag). While defined differently than group A *var* genes, the cys-2 *var* group correlates tightly with the group A *var* group, and the expression of group A *var* genes has been shown to correlate with severe malaria symptoms in multiple populations [12]. DBLα sequence tags containing other number of cysteines correlate with group B and C *var* types, and these have been associated with mild and/or asymptomatic malaria generally—although there are notable exceptions [12].

## Supporting information

S1 CodeAndData A compressed file containing all the code and data needed to run the main analysis of this study. The data is in the form of a CSV file with the homology block presence/absence state for each of the *var* types used in this study. The code files are written in R.
(ZIP)
